# Design of novel disturbing peptides against ACE2 SARS-CoV-2 spike-binding region by computational approaches

**DOI:** 10.3389/fphar.2022.996005

**Published:** 2022-11-11

**Authors:** Sara Zareei, Saeed Pourmand, Massoud Amanlou

**Affiliations:** ^1^ Department of Cell and Molecular Biology, Faculty of Biological Sciences, Kharazmi University, Tehran, Iran; ^2^ Department of Chemical Engineering, Faculty of Chemical and Petroleum Engineering, University of Tabriz, Tabriz, Iran; ^3^ Department of Medicinal Chemistry, Faculty of Pharmacy, Tehran University of Medical Sciences, Tehran, Iran; ^4^ Experimental Medicine Research Center, Tehran University of Medical Sciences, Tehran, Iran

**Keywords:** SARS-CoV-2, COVID-19, angiotensin converting enyzme 2, peptide design, molecular dynamics simulation, drug discovery

## Abstract

The SARS-CoV-2, the virus which is responsible for COVID-19 disease, employs its spike protein to recognize its receptor, angiotensin-converting enzyme 2 (ACE2), and subsequently enters the host cell. In this process, the receptor-binding domain (RBD) of the spike has an interface with the α1-helix of the peptidase domain (PD) of ACE2. This study focuses on the disruption of the protein-protein interaction (PPI) of RBD-ACE2. Among the residues in the template (which was extracted from the ACE2), those with unfavorable energies were selected for substitution by mutagenesis. As a result, a library of 140 peptide candidates was constructed and the binding affinity of each candidate was evaluated by molecular docking and molecular dynamics simulations against the α1-helix of ACE2. Finally, the most potent peptides P23 (GFNNYFPHQSYGFMPTNGVGY), P28 (GFNQYFPHQSYGFPPTNGVGY), and P31 (GFNRYFPHQSYGFCPTNGVGY) were selected and their dynamic behaviors were studied. The results showed peptide inhibitors increased the radius, surface accessible area, and overall mobility of residues of the protein. However, no significant alteration was seen in the key residues in the active site. Meanwhile, they can be proposed as promising agents against COVID-19 by suppressing the viral attachment and curbing the infection at its early stage. The designed peptides showed potency against beta, gamma, delta, and omicron variants of SARS-CoV-2.

## Introduction

Since the outbreak of the COVID-19 pandemic in 2020, extensive endeavors have been made to protect human lives against viral infection. Despite the steadily decreased number of infected cases following the wide vaccine distribution, the outbreaks are seen in regions with high vaccination rates (Lipsitch et al., 2022), challenging the termination of the pandemic. Moreover, some warn that the immunity which is expected to be provided by vaccines decreases after a while, and therefore, vaccines may not be fully reliable (Yewdell, 2021). While the booster doses are indeed administrated in several countries, the reports of medical problems after vaccination (Tinoco et al., 2021; Al-Midfai et al., 2022; Chen et al., 2022; Fathy et al., 2022; Sriwastava et al., 2022) alarm that the current vaccines may not be wholesome in the long term. Considering the aforementioned conditions, the development of novel specific anti-COVID19 therapeutics seems to be a compelling urge for pandemic eradication.

Today, there is no doubt that a glycoprotein protein, Spike (S) is responsible for host cell recognition and viral fusion by the SARS-COV-2 virus (Li, 2016; Wan et al., 2020). Spike protein (S) is a trimeric glycoprotein that mainly allows coronaviruses to target host cells (Bosch et al., 2003) by their receptor-binding domain (RBD). RBD recognizes and binds to angiotensin-converting enzyme 2 (ACE2) through which the virus can import its genetic material (Li, 2015; Scialo et al., 2020; Rezaei et al., 2021). ACE2 is a type I transmembrane protein with wide distribution in many tissues including testes, heart, kidney, liver, intestines, lungs, brain, and oral mucosa (Tipnis et al., 2000; Hamming et al., 2004; Paizis et al., 2005; Doobay et al., 2007; Fan et al., 2020; Xu et al., 2020) which underpins multi-organ manifestation of COVID-19.

ACE2 expands through the cellular membrane consisting of an N-terminal peptidase domain (PD, residues 19–615) and a collectrin-like transmembrane domain (616–726) (Yan et al., 2020) (Supplementary Figure S1). PD accommodates viral RBD and is engaged in viral entry (Lan et al., 2020; Shang et al., 2020). It serves in renin angiotensin aldosterone system (RAAS) as a hydrolase. ACE2 converts angiotensin II, which stimulates the contraction of blood vessels, to angiotensin 1–9, which are vasodilators. Therefore, ACE2 is a key enzyme in the blood pressure regulation system (Donoghue et al., 2000; Turner, 2015).

Two mechanisms are identified for SARS-CoV-2 pathogenesis following the attachment of the virus to PD. In the first which is called “endosomal entry,” the whole receptor-bound virus is embraced by an endosome where cathepsin L activates the S protein and enables it to mediate the fusion of membranes and subsequently cause viral RNA entry into the cytoplasm (Khan et al., 2020). The second mechanism, however, is initiated by S activation at the outer surface where the surface protease TMPRSS2 exists. Similar to endosome-mediated entry, this mechanism also relies on ACE2 recognition (Hoffmann et al., 2020).

Due to the vital role of ACE2 recognition in SARS-CoV-2 pathogenesis (Singh et al., 2021), this enzyme has drawn special attention as a therapeutic target (Jia et al., 2021) although the beneficial inhibition of ACE2 has been exposed to discussion against lung injury before the pandemic (Imai et al., 2005). In this regard, a variety of strategies have been applied with ACE2 as the main element. It has been demonstrated that engineered variants of ACE2 compete with native ACE2 to attach SARS-CoV-2 spike proteins, leaving the native form free to perform its natural role (Krishnamurthy et al., 2021). Moreover, an engineered form of ACE2 with a trimeric structure has also been applied against COVID-19 (Guo et al., 2021). In another strategy, RBD -ACE2 interaction was targeted by various inhibitors such as repurposed approved drugs (Choudhary et al., 2020; Teralı et al., 2020), peptides, and peptidomimetics (Dahal et al., 2022; Zhao et al., 2022).

Computational techniques and molecular dynamics simulations have been widely applied in COVID-19-related studies in different areas such as interactions between the virus and human its human receptors (Spinello et al., 2020; Wang et al., 2020), mutations of SARS-CoV-2 (Shah et al., 2020; Kullappan et al., 2021; Luan et al., 2021; Spinello et al., 2021), characteristics of SARS-CoV-2 proteins (Kordzadeh and Saadatabadi, 2021; Zhang et al., 2022; Bignon et al., 2022; Borišek et al., 2021), and drug discovery (Aallaei et al., 2021; Bayati and Ebrahimi, 2021; Mora et al., 2022). In our previous study, we designed peptide inhibitors with the last strategy inspired by the RBD domain of the SARS-CoV-2 spike (Pourmand et al., 2022). Here, computational mutagenesis was applied to designing new peptide inhibitors to provide physical disturbance against SARS-CoV-2 and ACE2 interaction inspired by the RBD-binding region of ACE2.

Since the outbreak of COVID-19, the virus has evolved into several new variants and sub-variants (Tao et al., 2021) which became dominant globally. The mutations that new variants bear endowed them with new features such as increased transmissibility, increased risk of reinfection, and/or reduced vaccine efficacy. Here, we studied the potential inhibitory effect of the designed peptides on several important variants of SARS-CoV-2 including Beta, Gamma, Delta, and Omicron.

## Computational methods

### Protein-protein interface analysis and template extraction

The peptide inhibitors were designed following the precise assessment of the key residues involved in the formation of the SARS-CoV-2-ACE2 interface similar to our previous study (Pourmand et al., 2022). The crystal structure of the RBD-ACE2 complex under the PDB ID of 6m0j was retrieved from the RCSB protein data bank in which the 3D structure of the Wuhan variant of SARS-CoV-2 in complex with ACE peptidase domain was crystallized. After monitoring the key residues in the RBD-PD interface by LigPlot^+^ (Laskowski and Swindells, 2011), RBD residues involved in ACE2 recognition and their surrounding residues (residues 485–505) were extracted as a template for peptide design.

### Peptide design

The possible mutations for each hotspot were performed by systematic mutation prediction in the mCSM server (http://biosig.unimelb.edu.au/mcsm) (Pires et al., 2014) which predicts the affinity change in the protein-protein complex providing ΔΔG for each site. The spike-ACE2 complex was uploaded and all mutation sites were introduced to the server. The residues with positive ΔΔG were given to OSPREY protein design software v. 3.0 to generate the mutant peptides library (Hallen et al., 2018).

### Peptide toxicity and stability assessment

The potential toxicity and allergenic activities of the mutant peptides were evaluated by the ToxinPred (https://webs.iiitd.edu.in/raghava/toxinpred/protein.php) and AllerTop (http://www.ddg-pharmfac.net/AllerTOP/) servers (Dimitrov et al., 2014).

### Molecular docking simulations

The safest peptides were docked to PD by the HADDOCK server (https://wenmr.science.uu.nl/haddock2.4/) (De Vries et al., 2010) and the complexes with the lowest binding scores were selected. HADDOCK is a fully automated server for protein-protein docking which needs receptor and peptide PDB files as input. All other parameters remained as default values. RBDs of variants Beta, Gamma, Delta, and Omicron were extracted from PDB IDs 7VX4, 7V84, 7WBQ, and 7WPC, respectively.

### Molecular dynamics simulations

To study the dynamical behaviors of template or chosen peptides, the strength of their interaction with the receptor, and to examine the overall stability of the complexes, MD simulations were conducted by GROMACS package version 2020 (Van Der Spoel et al., 2005) for 100 ns The receptor and peptides were parameterized using Gromos96 54a7 force field (Schmid et al., 2011). Then, each complex was put in a cubic box with a minimum distance of 1.0 nm from the edges. All systems were solvated in the simple-point charge (SPC) water model (Berendsen et al., 1987). The simulation boxes were neutralized by adding Na^+^ ions. Detailed information on all systems is given in Supplementary Table S1. Furthermore, all systems were energy minimized for 50,000 steps using the steepest descent method followed by a thermal equilibrium step (NVT) of 1ns using a Berendsen thermostat at 310 K. For pressure equilibration, the NPT step was carried out approximately for 1ns before the pressure of 1 bar was approached. LINCS algorithm was chosen for NVT, NPT, and production steps to restrain the bonds’ lengths (Hess et al., 1997). Furthermore, Particle Mesh Ewald (PME) was applied for the calculation of long-range electrostatic interactions and r-coulomb of 1.2 (Darden et al., 1993). Van der Waals interactions were also defined by Verlet using a cut-off value of 1.2. Finally, well-equilibrated systems underwent MD production for 100 ns.

The MD trajectories were analyzed using *gmx rms, rmsf, sasa*, *and gyrate* utilities to obtain the root mean square deviation (RMSD), root mean square fluctuation (RMSF), SASA (surface accessible solvent area), and radius of gyration (Rg), respectively. The number of hydrogen bonds were evaluated by *hbond* toolkit of GROMACS and their occupancy values were obtained by *readhbmap.py* script. In both methods of analysis, the hydrogen bonds were defined by distance less than 3.5A° and angle of 30°. Furthermore, dominant and collective motions of the protein were identified by principal component analysis (PCA) for the last 20 ns of simulations. Applying *gmx covar* tool, the covariance matrix was calculated and then diagonalized so that eigenvalues and eigenvectors were obtained. In the next step of PCA, *gmx anaeig* toolkit was utilized to provide 2D plot values. The total free energy binding, electrostatic, and van der Waals energies of each residue were obtained using the mmpbsa python script (Kumari et al., 2014) to detail the role of each residue located in the peptide-PD interface. This script works based on the MM/PBGBSA method (Genheden and Ryde, 2015) which calculates the binding free energies of complexes formed by non-covalent bonds. The movements of the protein’s subdomains were identified by modevectors.py (Sean, 2012) after the extraction of the initial and final frames of each simulation.

## Results and discussion

The present study mainly aimed at the design of novel peptide inhibitors against SARS-CoV-2 pathogenesis at the host recognition step during which the viral S protein binds to the host ACE2 peptidase domain, then the viral genetic material enters the cell and subsequently, the viral cycle initiates (Lan et al., 2020). Logically, we considered two necessities for designing the peptides. First, the inhibitors should have greater affinity than the RBD for ACE2 so that they can compete with the virus. Moreover, the natural catalytic activity of the peptide-bound ACE2 must remain intact to avert several conditions such as hypertension and kidney diseases in which ACE2 activity declined (Crackower et al., 2002; Zhong et al., 2004).

As made clear by X-ray differentiation, the SARS-CoV-2’s RBD residues Lys417, Gly446, Tyr449, Tyr453, Leu455, Phe456, Ala475, Phe486, Asn487, Tyr489, Gln493, Gly496, Gln498, Thr500, Asn501, Gly502, and Tyr505 lying at chain E of spike’s RBD domain were responsible for the virus’ attachment while Gln24, Thr27, Phe28, Asp30, Lys31, His34, Glu37, Asp38, Tyr41, Gln42, Leu79, Met82, Tyr83, Asn330, Lys353, Gly354, Asp355, and Arg357 of ACE2 enzyme are recognized by RBD (Lan et al., 2020) (Supplementary Figure S1). It has been shown that ACE2 residues Gln24, Thr27, Tyr83, and Lys353 are key stabilizing residues for the RBD-ACE2 adduct (Spinello et al., 2020). Therefore, we selected RBD residues 485–505 as a template for peptide design aimed to inhibit this region. Other studies used other fragments of S protein for peptide design (Fukushi et al., 2005; Hu et al., 2005; Ho et al., 2006; Struck et al., 2012; Han and Král, 2020; Panda et al., 2021).

**FIGURE 1:**
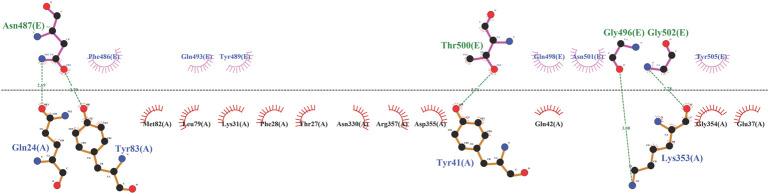
The illustration of interactions between PD (black) and template (blue).

To begin, the enzyme and template were docked to validate the simulation process. It can be seen in Figure 1 that the template lay in the cavity that RBD did. Asp30, His34, and Asp38 of ACE2 were not involved in template binding while they were seen among the interactions between RBD and the receptor (Supplementary Figure S1). However, the template made connections with the stabilizing residues (Gln24, Thr27, Tyr83, and Lys353) (Figure 1). In the next step, the template-PD complex undertook a 100-ns simulation to identify the contribution of each template residue to receptor binding by the mmpbsa method. This analysis affords the opportunity of determining the residues template residues with unfavorable energy and hence, their negative impact on the template’s binding to the receptor. Table 1 shows the results of mmpbsa analysis in which residues Cys488, Gln498, and Tyr505 had positive total energies, and Leu 492 had positive electrostatic energies suggesting their negative impact on the template’s ΔG_binding_. Therefore, they were replaced by amino acids with more negative ΔG to improve the binding properties of the inhibitors.

**TABLE 1:** The mmpbsa energy analysis of the template residues revealed by a 50-ns MD simulation.

Residue	Energy component (kJ/mol)		
	ΔE_vdw_	ΔE_ele_	ΔG_Total_
485GLY	−6.22806	−993.986	−1000.21
486 PHE	−30.1342	−8.98672	−39.1209
487ASN	−13.2629	−24.1255	−30.3884
488CYS	−6.49005	14.53162	8.04159
489TYR	−32.8491	−40.5409	−73.39
490PHE	−8.87016	−26.653	−35.5231
491PRO	−10.6615	−10.9168	−21.5784
492LEU	−27.2245	8.622336	−18.6022
493GLN	−11.5028	−26.7256	−38.2284
494SER	−5.85836	−113.279	−119.137
495TYR	−7.97721	−80.1352	−88.1124
496GLY	1.062713	−77.8582	−76.7955
497PHE	−27.0681	−74.7586	−101.827
498GLN	−7.68477	25.42485	17.74007
499PRO	−10.0018	−22.1107	−32.1124
500THR	−11.3576	−65.9389	−77.2965
501ASN	−6.18565	−9.99557	−16.1812
502GLY	−6.2698	−2.77311	−9.04293
503VAL	−30.0826	−3.95751	−34.0401
504GLY	−12.3298	−12.1222	−24.452
505TYR	−32.8888	983.8618	950.9729

Since hydrogen bonds play a vital role in protein-protein interaction (Salentin et al., 2014), we performed the hydrogen bond occupancy analysis. The results revealed that Tyr505 is capable of forming a strong connection with the receptor through its four hydrogen bonds compared to other hotspots (Table 2). This suggests that this residue positively contributes to template binding and was kept without replacement. In other studies, peptide libraries were constructed differently. For instance, Curreli et al. (2020) applied phage biopanning for mutating Gln24, Asp30, Glu35, Asp38, Tyr41, and Gln42 and their designed peptide experimentally proved to block SARS-CoV-2 infectivity. In another study, computational alanine screening was used to identify favorable residues for substitution in the peptide inhibitors whose inhibitory effects were shown *in vivo* (Shah et al., 2022). Following the identification of hot spots, amino acids that were capable of being located in each position and their ΔΔG values were obtained using mCSM sever (Table 3). Among them, 12 residues with positive energies were presented to Osprey leading to a 140-peptide library (Supplementary Table S2) among which 22 peptides were identified as allergically and toxically safe (Supplementary Table S3).

**TABLE 2:** The occupancy of H-bonds between the template peptide and ACE2 chain A.

Pair ID	Donor-acceptor	Occupancy (%)
1	505TYR (HH)—37 GLU(OE2)	31.3
2	505TYR (HH)—37 GLU(OE1)	41.8
3	501ASN(H)—353LYS(O)	14.6
4	500THR (HG1)—355ASP(OD2)	46.3
5	500THR (HG1)—355ASP(OD1)	46.2
6	500THR (H)—353LYS(O)	76.4
7	497PHE(H)—38 ASP(OD2)	16.6
8	497PHE(H)—38 ASP(OD1)	69.9
9	496GLY (H)—38 ASP(OD2)	29.3
10	496GLY (H)—38 ASP(OD1)	68.3
11	495TYR (H)—38 ASP(OD2)	71.1
12	495TYR (H)—38 ASP(OD1)	15.6
13	494SER(HG)—35 GLU(OE2)	56.8
14	494SER(HG)—35 GLU(OE1)	52.9
15	494SER(H)—35 GLU(OE2)	40
16	494SER(H)—35 GLU(OE1)	34
17	490PHE(H)—75 GLU(OE2)	33.2
18	490PHE(H)—75 GLU(OE1)	40.6
19	487ASN(D21)—24 GLN (O)	10.6
20	487ASN(H)—83 TYR (OH)	16
21	393ARG (H11)—505TYR (OH)	11.2
22	353LYS(HZ1)—497PHE(O)	33.2
23	353LYS(H)—505TYR (OH)	12.9
24	83TYR (HH)—487ASN(OD1)	14.5

**TABLE 3:** The hotspot mutation results from mCSM server.

Hotspot	Proper amino acids (ΔΔG)
Cys488	Ala (−0.467)
	Val (−0.336)
	Leu (−0.303)
	Gly (−0.564)
	Ser (−0.44)
	Trp (−0.573)
	Thr (0.028)
	Gln (0.254)
	Glu (0.416)
	Arg (0.477)
	Pro (−0.336)
	Asp (0.454)
	Phe (−0.566)
	Ile (−0.303)
	His (−0.363)
	Asn (0.377)
	Met (−0.044)
	Tyr (−0.399)
	Lys (0.135)
Leu492	Ala (−0.68)
	Val (−0.476)
	Gly (−0.819)
	Ser (−0.848)
	Trp (−0.683)
	Thr (−0.798)
	Gln (−0.576)
	Glu (−0.381)
	Cys (−0.903)
	Arg (0.016)
	Pro (−0.476)
	Asp (−0.324)
	Phe (−0.668)
	Ile (−0.415)
	His (0.011)
	Asn (−0.444)
	Met (−0.766)
	Tyr (−0.465)
	Lys (−0.462)
Gln498	Ala (−0.749)
	Val (−0.164)
	Leu (0.085)
	Gly (−1.077)
	Ser (−0.88)
	Trp (−0.369)
	Thr (−0.567)
	Glu (0.12)
	Cys (−0.077)
	Arg (−0.013)
	Pro (−0.164)
	Asp (0.014)
	Phe (−0.522)
	Ile (0.085)
	His (−0.609)
	Asn (−0.698)
	Met (0.35)
	Tyr (−0.157)
	Lys (−0.112)

**FIGURE 2:**
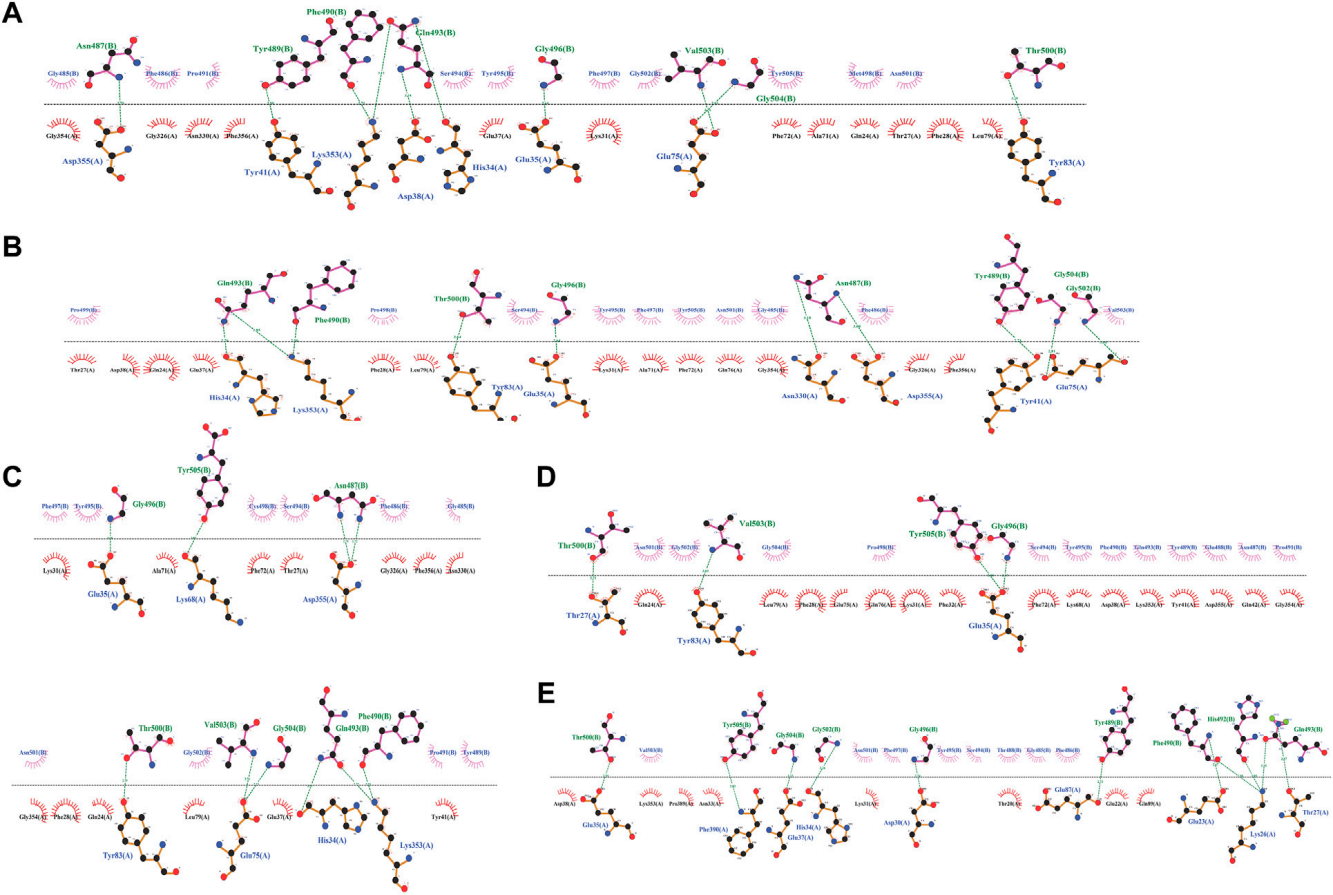
The detailed interactions between peptide inhibitors P23, P28, P31, P4, and P68 **(A-E)**.

Safe candidates were docked against PD. The results showed that P23, P31, and P28 had the lowest binding scores, and hence the highest affinities against ACE2, with values of -121, -117, and -114, respectively (Supplementary Table S3). Figure 2 details the interactions in the PD-peptides interface. It can be seen that Lys86 was only involved in the P31 interface while Asp38 was seen in P28 and P23 interfaces (Figures 2A–C). This suggests these residues may have a strengthening/weakening role in the inhibitory potential of these peptides, respectively. Furthermore, three key residues for SARS-CoV-2 infection, namely Gln24, Thr27, and Tyr83, are engaged in peptides’ binding. This suggests that the inhibitors may prevent the virus from recognizing its suitable location for causing infection (Spinello et al., 2020).

At the other extreme, P68 and P4 showed the lowest docking scores of 104 and 105, respectively (Supplementary Table S3). As Supplementary Figure S2A illustrates, these peptides also lay at the position where SARS-CoV-2 starts its recognition step. However, their binding was made by ACE2 residues Thr20, Glu22, Glu23, Lys 26, Asp30, Phe32, Asn33, Glu42, Lys68, Glu87, Gln89, and Pro389 which showed no bond with P23, P28, and P31. On the other hand, Ala71, Gly326, Asn330, Gly326, and Phe356 are connected exclusively with the most potent inhibitors’ binding. This matter may suggest the decreasing/increasing impact of PD on the potencies of the peptides, respectively (Figures 2D,E). Regarding peptides’ structure, such different potencies are rooted in their sequences in positions 4 and 14 (Supplementary Figure S2A). P28, P4, and P68 had an aromatic (proline) residue in position 14. Comparing these peptides, we can see that a bulkier residue in position 4 can improve the potency from P68 (with Thr) to P4 (with Glu). Moreover, the amide functional group also enhanced the peptide affinity in P28 compared to P4. When aliphatic residues lie in position 14, like P23 and P43, a bulkier residue in position 14 (Met compared to Cys) and a smaller one in position 4 (Asn compared to Arg) enhance the peptide’s affinity (Supplementary Figure S2B). The substitution of Asn and Met in positions 4 and 14 decreased Rg and SASA of P23 suggesting that this peptide adopts a more condensed structure compared to P28 and P31 (Supplementary Figures S3A,B). Moreover, Met and Cys in P23 and P31 were the least flexible residues (Supplementary Figure S3C).

To make sure that the RBD domain of SARS-CoV-2 becomes unable to recognize its favorable site for binding, and hence trigger the infection process, we conducted control molecular docking simulations in which P23, P28, and P31-bound ACE2 were docked against RBD. As can be seen in Supplementary Figure S4, RBD failed to locate its specific region of pathogenesis. Otherwise, other parts of RBD made a connection with ACE2, not the RBM motif (Supplementary Figure S4). Docking simulations of the peptides with Beta, Gamma, Delta, and Omicron RBDs showed that P23 and P28 impeded the formation of the RBD-PD complex (Supplementary Figures S5A,B). P31, however, failed to hinder the binding of RBD_Beta_ and hence may have a lower efficiency against the Beta variant (Supplementary Figure S5C).

**FIGURE 3:**
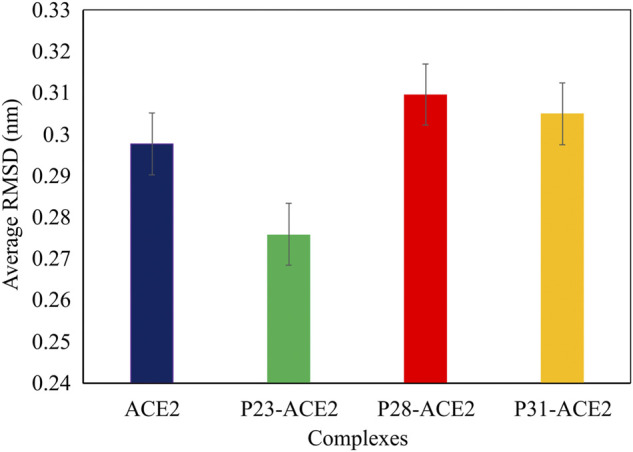
The average RMSD values of αcarbons of PD domain of ACE in apo (dark blue) and in complex with P23 (green), P28 (red), P31 (orange).

**FIGURE 4:**
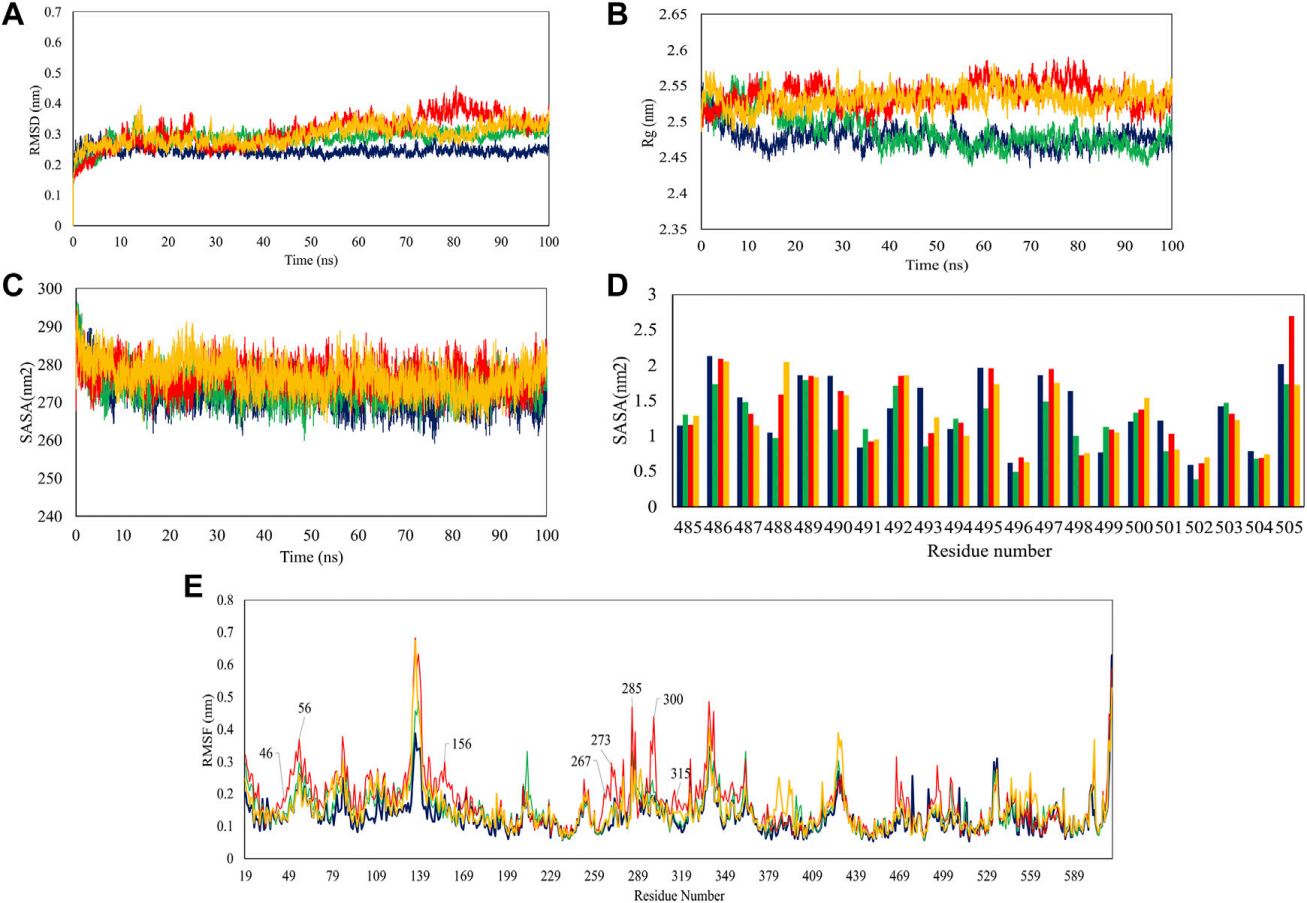
Trajectory analysis of 100-ns simulations in terms of RMSD **(A)**, Rg **(B)**, overall SASA **(C)**, residue-based SASA **(D)**, and RMSF **(E)**. PD is depicted as violet while its complex with P23, P28, and P31 are shown in green, red, and orange, respectively.

**FIGURE 5:**
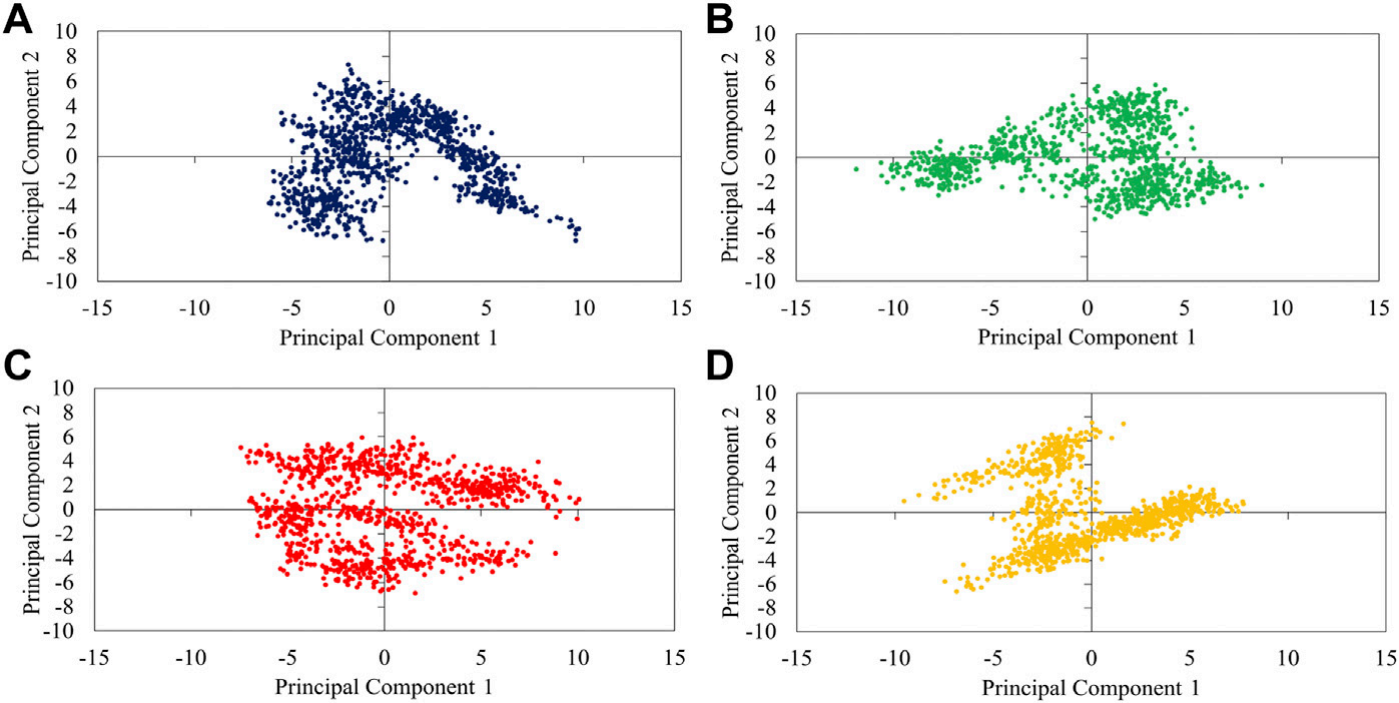
2D-projection of protein motions along the first (PC1) and second (PC2) principal components for free PD **(A)** and bound with P23 **(B)**, P28 **(C)**, and P31 **(D)**.

In the next step, we evaluated the dynamic behaviors of free and peptide-bound PD by molecular dynamics simulations (Figures 3–5). RMSD analysis measures the difference between Cα atoms of ACE2 in each simulation frame and the initial frame. The minimum and maximum average RMSD values belonged to P23 (0.28 nm) and P28 (0.30 nm) (Figure 3) suggesting the minimum and maximum structural deviation and hence conformational changes these peptides induce in PD. Since drastic conformational changes may destabilize PD and affect its catalytic activity, we plotted the RMSD values *versus* time (Figure 4A). All complexes reached equilibrium at the initial 10 ns of the simulation. P31- and P23-bound PD underwent no drastic fluctuations implying the stability of these complexes. However, P28 induced a significant change in the protein’s conformation after 75 ns.

To see how large these changes might be, Rg, and SASA analyzes were carried out because significant changes can affect the radius of the protein and its access to the solvent. The results showed that P28 and P31 revealed PD structure to the solvent as their higher Rg values suggest. P23, however, ended up at Rg close to apo-PD after 20 ns (Figure 4B). Rg results are reflected in SASA analysis in which peptides increased protein’s accessible area to the solvent except for P23 (Figure 4C). Regarding the inhibitors, residue-based SASA analysis consistently showed that most peptide residues with hydrogen bonds to the receptor, including Asn487, Gln493, Gly496, and Gly504 had lower SASA and therefore, had smaller exposure to solvent (Figure 4D). Based on the result, P23 can be considered the least exposed inhibitor suggesting that this inhibitor might be successful in fitting with the external cavity of PD which explains its higher affinity score.

According to RMSD and Rg results, when peptide inhibitors are bound to the receptor, they can alter the protein’s features for which conformational changes are required. To understand which residues are responsible for such actions and whether active site residues are also included, we used RMSF analysis which measures the gratitude of movements in protein residues in a timeline. As Figure 4E illustrates, P23 and P31 had similar peaks and troughs to the protein although with greater fluctuations. This suggests that peptide inhibitors increased the mobility of flexible regions but did not make the rigid parts flexible. However, P28 showed a significant increase in several regions including the residues in positions 46–56, 156, 267–273, 299–300, and 315. Taking Rg results into account (Figure 4B), it can be deduced that the high mobility of these regions might be responsible for the greater volume of the receptor which was reflected in higher Rg values.

The analysis of overall collective motions by which protein may assume new conformations showed that P28 and P31 induced/limited collective motions in PD since they covered greater/smaller conformational space compared to apo-protein, respectively (Figures 5C,D). P23 had as great expansion as apo-PD but in the opposite direction.

Due to the importance of hydrogen bonding in target recognition and the stability of a protein-protein complex (Hubbard and Haider, 2010), we studied the creation and breakage of hydrogen bonds involved in the peptides-PD interface. According to Supplementary Figure S6, dynamic hydrogen bonds between rationally designed peptides and PD contributed to the relative stability of the systems. The residue-based analysis also indicated that P23 and P31 established a stronger (as higher occupancy values suggest) higher number of dynamic H-bonds which is another reason for their higher potency against template and P28 (Supplementary Table S4). Moreover, mmpbsa analysis showed that electrostatic and Van der Waal energies played important roles in peptide binding (Table 4).

**TABLE 4:** The mmpbsa analysis of designed peptides’ binding.

Energy	P23	P28	P31
Van der Waal energy	−247.343 ± 25.496	−158.811 ± 7.259	−192.897 ± 22.558
Electrostatic energy	−935.105 ± 27.011	−295.134 ± 23.813	−796.565 ± 71.079
Polar solvation energy	1098.955 ± 25.680	377.392 ± 33.661	912.369 ± 103.254
SASA energy	−38.414 ± 1.765	−27.640 ± 2.036	−37.145 ± 1.942
Binding energy	−121.907 ± 22.987	−104.193 ± 18.092	−114.238 ± 57.578

**FIGURE 6:**
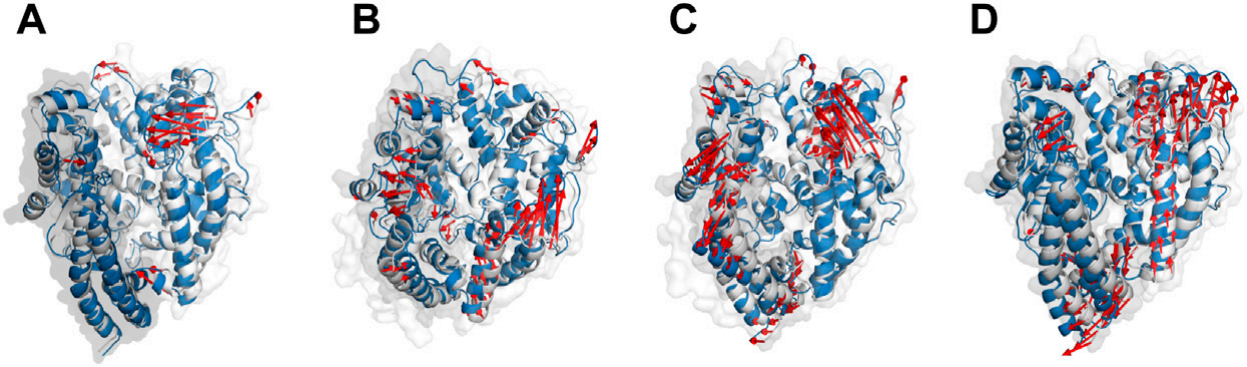
The main motion of PD **(A)**, P23-PD **(B)**, P28-PD **(C)** and P31-PD **(D)** during the initial (white cartoon) and the last (blue cartoon) frames of the simulations. The subdomains I and II are represented as grey and white transparent cartoons, respectively.

In addition to the safety analysis (Supplementary Table S2), we seek to check whether the designed peptides may have a detrimental effect on the body by inhibiting PD’s catalytic activity and hence the vital physiological function that ACE2 plays (Turner, 2015). The active site of PD is recessed at the bottom of a 40°A-long cleft which is formed by PD’s subdomains I and II (sub I and sub II). Before ligand binding, both subdomains adopt an “open” conformation by which the active site is revealed to the environment. Inhibitors such as MLN-4760, however, induce “close” conformation in which sub I move toward the relatively stable subII leading to the deepening of the active site (Towler et al., 2004). The receptor’s cleft becomes partially open when SARS-CoV-2 RBD settles on ACE2 (Gross et al., 2020). We found that none of the key residues in ACE2’s active site, His374, Glu375, His 378, Glu402, Glu406, His505, and Tyr515 (Guy et al., 2003), were inhibited by peptides (Figure 2). Conformationally, apo-PD showed subtle motions in sub I while sub II had greater motions (Figure 6A). Inhibitors’ binding, however, induced greater movements in both domains compared to the apo state. P23 induced open conformation in sub I which may suggest inducing higher catalytic activity of the active site that has been exposed to the environment (Figure 6B). Sub II became more flexible upon binding of all inhibitors in a similar direction to ACE2 (Figures 6C,D). These results show that P23 may be a weak activator of ACE2 while other peptides may cause nuance in the peptidase activity of ACE2.

P4 and P68 behaved differently compared to the top hits during the simulation. Both of them had significantly greater RMSD, Rg, and SASA suggesting greater changes they induce in PD conformation (Supplementary Figure S7). Moreover, while P23, 28, and 31 increased the mobility of a few mostly outer residues of both subdomain I and II, P4 and 68 showed an extensive change in many residues especially those located at the protein’s core in subdomain II (Supplementary Figure S8). Therefore, inferior peptides’ on the ACE2 conformation may be more intense.

## Conclusion

Our study proposes potential peptide inhibitors that may inhibit SARS-CoV-2 infection by protecting its host receptor, ACE2. Rationally designed peptides were computationally assessed in terms of safety, affinity, and dynamic behavior. The results showed that P23 (GFNNYFPHQSYGFMPTNGVGY), P28 (GFNQYFPHQSYGFPPTNGVGY), and P31 (GFNRYFPHQSYGFCPTNGVGY) may be considered lead peptides for anti-COVID-19 peptide agents. Since SARS-CoV-2’s binding site does not overlap with the ACE2 active site, designed peptides are capable of inhibiting SARS-CoV-2 infection, either caused by Wuhan strain or beta, gamma, delta, and omicron variants, without any probable consequence for the normal function of ACE2.

## Data Availability

The original contributions presented in the study are included in the article/Supplementary Materials, further inquiries can be directed to the corresponding author.
