# Supplementary material for: Design of novel disturbing peptides against ACE2 SARS-CoV-2 spike-binding region by computational approaches
Source: Front Pharmacol. 2022 Nov 11;13:996005. doi: 10.3389/fphar.2022.996005 (PMC9692113; doi:10.3389/fphar.2022.996005)
Supplement: Supplementary file 1 [file Table1.DOCX]

Design of Novel Disturbing Peptides Against ACE2 SARS-CoV-2 Spike-binding Region by Computational Approaches

Sara Zareei ^1†^, Saeed Pourmand^2^ ^†^, Massoud Amanlou^3,4*^

^1^ Department of Cell & Molecular Biology, Faculty of Biological Sciences, Kharazmi University, Tehran, Iran

^2^ Department of Chemical Engineering, Faculty of Chemical and Petroleum Engineering, University of Tabriz, Tabriz, Iran

^3^ Department of Medicinal Chemistry, Faculty of Pharmacy, Tehran University of Medical Sciences, Tehran, Iran

^4^ Experimental Medicine Research Center, Tehran University of Medical Sciences, Tehran, Iran

Supplementary Material

# Supplementary Table

| Table S1- Summary of simulations including the molecules, ions, waters, and box size. | | | |
| --- | --- | --- | --- |
| Systems | No. of Ions | No. of Waters | Box Size (nm) |
| 23-PD | 28Na^+^ | 29023 | 9.91 × 9.91 × 9.91 |
| 28-PD | 28Na^+^ | 29957 | 9.98 × 9.98 × 9.98 |
| 31-PD | 27Na^+^ | 30710 | 10.08 × 10.08 × 10.08 |
| 4-PD | 29Na^+^ | 31196 | 10.12 × 10.12 × 10.12 |
| 68-PD | 28Na^+^ | 32095 | 10.21 × 10.21 × 10.21 |
| Template-PD | 27Na^+^ | 34656 | 10.45 × 10.45 × 10.45 |

| Table S2. the 144-peptide library after mutation and biocompatibility analysis. | | |
| --- | --- | --- |
| No. | **Peptide Sequence** | **Safety and Allergenicity Potential** |
| 1 | GFNTYFPHQSYGFVPTNGVGY | PROBABLE ALLERGEN |
| 2 | GFNDYFPHQSYGFRPTNGVGY | PROBABLE ALLERGEN |
| 3 | GFNQYFPHQSYGFLPTNGVGY | PROBABLE ALLERGEN |
| 4 | GFNEYFPHQSYGFPPTNGVGY | PROBABLE NON-ALLERGEN |
| 5 | GFNNYFPHQSYGFDPTNGVGY | PROBABLE ALLERGEN |
| 6 | GFNRYFPHQSYGFEPTNGVGY | PROBABLE ALLERGEN |
| 7 | GFNKYFPHQSYGFCPTNGVGY | PROBABLE NON-ALLERGEN |
| 9 | GFNTYFPHQSYGFIPTNGVGY | PROBABLE ALLERGEN |
| 10 | GFNDYFPHQSYGFMPTNGVGY | PROBABLE NON-ALLERGEN |
| 11 | GFNQYFPHQSYGFKPTNGVGY | PROBABLE ALLERGEN |
| 12 | GFNEYFPHQSYGFVPTNGVGY | PROBABLE ALLERGEN |
| 13 | GFNNYFPHQSYGFRPTNGVGY | PROBABLE ALLERGEN |
| 14 | GFNRYFPHQSYGFLPTNGVGY | PROBABLE ALLERGEN |
| 15 | GFNKYFPHQSYGFPPTNGVGY | PROBABLE NON-ALLERGEN |
| 17 | GFNTYFPHQSYGFDPTNGVGY | PROBABLE ALLERGEN |
| 19 | GFNDYFPHQSYGFEPTNGVGY | PROBABLE ALLERGEN |
| 20 | GFNQYFPHQSYGFCPTNGVGY | PROBABLE NON-ALLERGEN |
| 22 | GFNEYFPHQSYGFIPTNGVGY | PROBABLE ALLERGEN |
| 23 | GFNNYFPHQSYGFMPTNGVGY | PROBABLE NON-ALLERGEN |
| 24 | GFNRYFPHQSYGFKPTNGVGY | PROBABLE ALLERGEN |
| 25 | GFNKYFPHQSYGFVPTNGVGY | PROBABLE ALLERGEN |
| 26 | GFNTYFPHQSYGFRPTNGVGY | PROBABLE ALLERGEN |
| 27 | GFNDYFPHQSYGFLPTNGVGY | PROBABLE ALLERGEN |
| 28 | GFNQYFPHQSYGFPPTNGVGY | PROBABLE NON-ALLERGEN |
| 29 | GFNEYFPHQSYGFDPTNGVGY | PROBABLE ALLERGEN |
| 30 | GFNNYFPHQSYGFEPTNGVGY | PROBABLE ALLERGEN |
| 31 | GFNRYFPHQSYGFCPTNGVGY | PROBABLE NON-ALLERGEN |
| 32 | GFNKYFPHQSYGFIPTNGVGY | PROBABLE ALLERGEN |
| 33 | GFNTYFPHQSYGFMPTNGVGY | PROBABLE NON-ALLERGEN |
| 34 | GFNDYFPHQSYGFKPTNGVGY | PROBABLE ALLERGEN |
| 35 | GFNQYFPHQSYGFVPTNGVGY | PROBABLE ALLERGEN |
| 36 | GFNEYFPHQSYGFRPTNGVGY | PROBABLE ALLERGEN |
| 37 | GFNNYFPHQSYGFLPTNGVGY | PROBABLE ALLERGEN |
| 38 | GFNRYFPHQSYGFPPTNGVGY | PROBABLE ALLERGEN |
| 39 | GFNKYFPHQSYGFDPTNGVGY | PROBABLE ALLERGEN |
| 40 | GFNTYFPHQSYGFEPTNGVGY | PROBABLE ALLERGEN |
| 41 | GFNDYFPHQSYGFCPTNGVGY | PROBABLE NON-ALLERGEN |
| 42 | GFNQYFPHQSYGFIPTNGVGY | PROBABLE ALLERGEN |
| 43 | GFNEYFPHQSYGFMPTNGVGY | PROBABLE NON-ALLERGEN |
| 44 | GFNNYFPHQSYGFKPTNGVGY | PROBABLE ALLERGEN |
| 45 | GFNRYFPHQSYGFVPTNGVGY | PROBABLE ALLERGEN |
| 46 | GFNKYFPHQSYGFRPTNGVGY | PROBABLE ALLERGEN |
| 47 | GFNTYFPHQSYGFLPTNGVGY | PROBABLE ALLERGEN |
| 48 | GFNDYFPHQSYGFPPTNGVGY | PROBABLE NON-ALLERGEN |
| 49 | GFNQYFPHQSYGFDPTNGVGY | PROBABLE ALLERGEN |
| 50 | GFNEYFPHQSYGFEPTNGVGY | PROBABLE ALLERGEN |
| 51 | GFNNYFPHQSYGFCPTNGVGY | PROBABLE NON-ALLERGEN |
| 52 | GFNRYFPHQSYGFIPTNGVGY | PROBABLE ALLERGEN |
| 53 | GFNKYFPHQSYGFMPTNGVGY | PROBABLE ALLERGEN |
| 54 | GFNTYFPHQSYGFKPTNGVGY | PROBABLE ALLERGEN |
| 55 | GFNDYFPHQSYGFVPTNGVGY | PROBABLE ALLERGEN |
| 56 | GFNQYFPHQSYGFRPTNGVGY | PROBABLE ALLERGEN |
| 57 | GFNEYFPHQSYGFLPTNGVGY | PROBABLE ALLERGEN |
| 58 | GFNNYFPHQSYGFPPTNGVGY | PROBABLE NON-ALLERGEN |
| 59 | GFNRYFPHQSYGFDPTNGVGY | PROBABLE ALLERGEN |
| 60 | GFNKYFPHQSYGFEPTNGVGY | PROBABLE ALLERGEN |
| 61 | GFNTYFPHQSYGFCPTNGVGY | PROBABLE NON-ALLERGEN |
| 62 | GFNDYFPHQSYGFIPTNGVGY | PROBABLE ALLERGEN |
| 63 | GFNQYFPHQSYGFMPTNGVGY | PROBABLE ALLERGEN |
| 64 | GFNEYFPHQSYGFKPTNGVGY | PROBABLE ALLERGEN |
| 65 | GFNNYFPHQSYGFVPTNGVGY | PROBABLE ALLERGEN |
| 66 | GFNRYFPHQSYGFRPTNGVGY | PROBABLE ALLERGEN |
| 67 | GFNKYFPHQSYGFLPTNGVGY | PROBABLE ALLERGEN |
| 68 | GFNTYFPHQSYGFPPTNGVGY | PROBABLE NON-ALLERGEN |
| 69 | GFNDYFPHQSYGFDPTNGVGY | PROBABLE NON-ALLERGEN |
| 70 | GFNQYFPHQSYGFEPTNGVGY | PROBABLE ALLERGEN |
| 71 | GFNEYFPHQSYGFCPTNGVGY | PROBABLE ALLERGEN |
| 72 | GFNNYFPHQSYGFIPTNGVGY | PROBABLE ALLERGEN |
| 73 | GFNRYFPHQSYGFMPTNGVGY | PROBABLE ALLERGEN |
| 74 | GFNKYFPHQSYGFKPTNGVGY | PROBABLE ALLERGEN |
| 75 | GFNTYFPRQSYGFVPTNGVGY | PROBABLE ALLERGEN |
| 76 | GFNDYFPRQSYGFRPTNGVGY | PROBABLE ALLERGEN |
| 77 | GFNQYFPRQSYGFLPTNGVGY | PROBABLE ALLERGEN |
| 78 | GFNEYFPRQSYGFPPTNGVGY | PROBABLE NON-ALLERGEN |
| 79 | GFNNYFPRQSYGFDPTNGVGY | PROBABLE ALLERGEN |
| 80 | GFNRYFPRQSYGFEPTNGVGY | PROBABLE ALLERGEN |
| 81 | GFNKYFPRQSYGFCPTNGVGY | PROBABLE ALLERGEN |
| 82 | GFNTYFPRQSYGFIPTNGVGY | PROBABLE ALLERGEN |
| 83 | GFNDYFPRQSYGFMPTNGVGY | PROBABLE ALLERGEN |
| 84 | GFNQYFPRQSYGFKPTNGVGY | PROBABLE ALLERGEN |
| 85 | GFNEYFPRQSYGFVPTNGVGY | PROBABLE ALLERGEN |
| 86 | GFNNYFPRQSYGFRPTNGVGY | PROBABLE ALLERGEN |
| 87 | GFNRYFPRQSYGFLPTNGVGY | PROBABLE ALLERGEN |
| 88 | GFNKYFPRQSYGFPPTNGVGY | PROBABLE ALLERGEN |
| 89 | GFNTYFPRQSYGFDPTNGVGY | PROBABLE ALLERGEN |
| 90 | GFNDYFPRQSYGFEPTNGVGY | PROBABLE ALLERGEN |
| 91 | GFNQYFPRQSYGFCPTNGVGY | PROBABLE ALLERGEN |
| 92 | GFNEYFPRQSYGFIPTNGVGY | PROBABLE ALLERGEN |
| 93 | GFNNYFPRQSYGFMPTNGVGY | PROBABLE ALLERGEN |
| 94 | GFNRYFPRQSYGFKPTNGVGY | PROBABLE ALLERGEN |
| 95 | GFNKYFPRQSYGFVPTNGVGY | PROBABLE ALLERGEN |
| 96 | GFNTYFPRQSYGFRPTNGVGY | PROBABLE ALLERGEN |
| 97 | GFNDYFPRQSYGFLPTNGVGY | PROBABLE ALLERGEN |
| 98 | GFNQYFPRQSYGFPPTNGVGY | PROBABLE NON-ALLERGEN |
| 99 | GFNEYFPRQSYGFDPTNGVGY | PROBABLE ALLERGEN |
| 100 | GFNNYFPRQSYGFEPTNGVGY | PROBABLE ALLERGEN |
| 101 | GFNRYFPRQSYGFCPTNGVGY | PROBABLE ALLERGEN |
| 102 | GFNKYFPRQSYGFIPTNGVGY | PROBABLE ALLERGEN |
| 103 | GFNTYFPRQSYGFMPTNGVGY | PROBABLE ALLERGEN |
| 104 | GFNDYFPRQSYGFKPTNGVGY | PROBABLE ALLERGEN |
| 105 | GFNQYFPRQSYGFVPTNGVGY | PROBABLE ALLERGEN |
| 106 | GFNEYFPRQSYGFRPTNGVGY | PROBABLE ALLERGEN |
| 107 | GFNNYFPRQSYGFLPTNGVGY | PROBABLE ALLERGEN |
| 108 | GFNRYFPRQSYGFPPTNGVGY | PROBABLE ALLERGEN |
| 109 | GFNKYFPRQSYGFDPTNGVGY | PROBABLE ALLERGEN |
| 110 | GFNTYFPRQSYGFEPTNGVGY | PROBABLE ALLERGEN |
| 111 | GFNDYFPRQSYGFCPTNGVGY | PROBABLE ALLERGEN |
| 112 | GFNQYFPRQSYGFIPTNGVGY | PROBABLE ALLERGEN |
| 113 | GFNEYFPRQSYGFMPTNGVGY | PROBABLE ALLERGEN |
| 114 | GFNNYFPRQSYGFKPTNGVGY | PROBABLE ALLERGEN |
| 115 | GFNRYFPRQSYGFVPTNGVGY | PROBABLE ALLERGEN |
| 116 | GFNKYFPRQSYGFRPTNGVGY | PROBABLE ALLERGEN |
| 117 | GFNTYFPRQSYGFLPTNGVGY | PROBABLE ALLERGEN |
| 118 | GFNDYFPRQSYGFPPTNGVGY | PROBABLE NON-ALLERGEN |
| 119 | GFNQYFPRQSYGFDPTNGVGY | PROBABLE ALLERGEN |
| 120 | GFNEYFPRQSYGFEPTNGVGY | PROBABLE ALLERGEN |
| 121 | GFNNYFPRQSYGFCPTNGVGY | PROBABLE ALLERGEN |
| 122 | GFNRYFPRQSYGFIPTNGVGY | PROBABLE ALLERGEN |
| 123 | GFNKYFPRQSYGFMPTNGVGY | PROBABLE ALLERGEN |
| 124 | GFNTYFPRQSYGFKPTNGVGY | PROBABLE ALLERGEN |
| 125 | GFNDYFPRQSYGFVPTNGVGY | PROBABLE ALLERGEN |
| 126 | GFNQYFPRQSYGFRPTNGVGY | PROBABLE ALLERGEN |
| 127 | GFNEYFPRQSYGFLPTNGVGY | PROBABLE NON-ALLERGEN |
| 128 | GFNNYFPRQSYGFPPTNGVGY | PROBABLE NON-ALLERGEN |
| 129 | GFNRYFPRQSYGFDPTNGVGY | PROBABLE ALLERGEN |
| 130 | GFNKYFPRQSYGFEPTNGVGY | PROBABLE ALLERGEN |
| 131 | GFNTYFPRQSYGFCPTNGVGY | PROBABLE ALLERGEN |
| 132 | GFNDYFPRQSYGFIPTNGVGY | PROBABLE ALLERGEN |
| 133 | GFNQYFPRQSYGFMPTNGVGY | PROBABLE ALLERGEN |
| 134 | GFNEYFPRQSYGFKPTNGVGY | PROBABLE ALLERGEN |
| 135 | GFNNYFPRQSYGFVPTNGVGY | PROBABLE ALLERGEN |
| 136 | GFNRYFPRQSYGFRPTNGVGY | PROBABLE ALLERGEN |
| 137 | GFNKYFPRQSYGFLPTNGVGY | PROBABLE ALLERGEN |
| 138 | GFNTYFPRQSYGFPPTNGVGY | PROBABLE NON-ALLERGEN |
| 139 | GFNDYFPRQSYGFDPTNGVGY | PROBABLE ALLERGEN |
| 140 | GFNQYFPRQSYGFEPTNGVGY | PROBABLE ALLERGEN |
| 141 | GFNEYFPRQSYGFCPTNGVGY | PROBABLE ALLERGEN |
| 142 | GFNNYFPRQSYGFIPTNGVGY | PROBABLE ALLERGEN |
| 143 | GFNRYFPRQSYGFMPTNGVGY | PROBABLE ALLERGEN |
| 144 | GFNKYFPRQSYGFKPTNGVGY | PROBABLE ALLERGEN |

| Table S3- Sequence of Safe Peptide Inhibitors derived from the Mutation of the Hotspots and their docking scores. | | |
| --- | --- | --- |
| Peptide No | **Peptide Sequence** | **Docking Score** |
| 4 | GFNEYFPHQSYGFPPTNGVGY | -105.9 ± 2.5 |
| 7 | GFNKYFPHQSYGFCPTNGVGY | -111.9 ± 3.8 |
| 10 | GFNDYFPHQSYGFMPTNGVGY | -106.9 ± 4.3 |
| 15 | GFNKYFPHQSYGFPPTNGVGY | -108.5 ± 2.1 |
| 20 | GFNQYFPHQSYGFCPTNGVGY | -111.6 ± 1.3 |
| 23 | GFNNYFPHQSYGFMPTNGVGY | -121.2 ± 2.1 |
| 28 | GFNQYFPHQSYGFPPTNGVGY | -114.3 ± 9.2 |
| 31 | GFNRYFPHQSYGFCPTNGVGY | -117.1 ± 5.9 |
| 33 | GFNTYFPHQSYGFMPTNGVGY | -107.8 ± 1.1 |
| 41 | GFNDYFPHQSYGFCPTNGVGY | -110.0 ± 3.8 |
| 43 | GFNEYFPHQSYGFMPTNGVGY | -106.0 ± 2.0 |
| 48 | GFNDYFPHQSYGFPPTNGVGY | -110.2 ± 3.5 |
| 51 | GFNNYFPHQSYGFCPTNGVGY | -112.8 ± 2.7 |
| 58 | GFNNYFPHQSYGFPPTNGVGY | -113.8 ± 4.4 |
| 61 | GFNTYFPHQSYGFCPTNGVGY | -108.1 ± 1.6 |
| 68 | GFNTYFPHQSYGFPPTNGVGY | -104.9 ± 3.7 |
| 69 | GFNDYFPHQSYGFDPTNGVGY | -111.5 ± 4.9 |
| 78 | GFNEYFPRQSYGFPPTNGVGY | -108.7 ± 5.4 |
| 98 | GFNQYFPRQSYGFPPTNGVGY | -112.3 ± 6.3 |
| 118 | GFNDYFPRQSYGFPPTNGVGY | -106.3 ± 3.6 |
| 127 | GFNEYFPRQSYGFLPTNGVGY | -108.2 ± 3.6 |
| 128 | GFNNYFPRQSYGFPPTNGVGY | -107.0 ± 2.9 |
| 138 | GFNTYFPRQSYGFPPTNGVGY | -107.8 ± 5.5 |

| Table S4- Comparison of H-bond occupancy between peptides P23, P28, and P31 and PD. | | | | | | | | |
| --- | --- | --- | --- | --- | --- | --- | --- | --- |
| P23-PD | | | P28-PD | | | P31-PD | | |
| Pair ID | donor-acceptor | Occupancy (%) | Pair ID | donor-acceptor | Occupancy (%) | Pair ID | donor-acceptor | Occupancy (%) |
| 1 | 505TYR(HH ) - 35 GLU(OE2) | 47.8 | 1 | 506TYR(HH ) - 25 GLN(OE1) | 14.4 | 1 | 505TYR(HH ) - 35 GLU(OE2) | 60.5 |
| 2 | 505TYR(HH ) - 35 GLU(OE1) | 46.3 | 2 | 505GLY( H ) - 84 TYR(OH ) | 10.9 | 2 | 505TYR(HH ) - 35 GLU(OE1) | 30.4 |
| 3 | 504GLY( H ) - 75 GLU(OE2) | 14.1 | 3 | 502ASN(D21) - 84 TYR(OH ) | 13.9 | 3 | 504GLY( H ) - 75 GLU(OE2) | 39.5 |
| 4 | 504GLY( H ) - 75 GLU(OE1) | 17.4 | 4 | 498PHE( H ) - 36 GLU(OE2) | 26.1 | 4 | 503VAL( H ) - 75 GLU(OE2) | 16.5 |
| 5 | 503VAL( H ) - 75 GLU(OE2) | 17.1 | 5 | 498PHE( H ) - 36 GLU(OE1) | 29.5 | 5 | 503VAL( H ) - 75 GLU(OE1) | 47.1 |
| 6 | 503VAL( H ) - 75 GLU(OE1) | 12.5 | 6 | 497GLY( H ) - 36 GLU(OE2) | 41 | 6 | 502GLY( H ) - 75 GLU(OE2) | 44.8 |
| 7 | 502GLY( H ) - 75 GLU(OE2) | 18.9 | 7 | 497GLY( H ) - 36 GLU(OE1) | 36.3 | 7 | 502GLY( H ) - 75 GLU(OE1) | 34.2 |
| 8 | 502GLY( H ) - 75 GLU(OE1) | 20.4 | 8 | 496TYR(HH ) - 39 ASP(OD2) | 13.9 | 8 | 501ASN(D21) - 76 GLN(OE1) | 50 |
| 9 | 501ASN(D21) - 76 GLN(OE1) | 89.1 | 9 | 496TYR(HH ) - 39 ASP(OD1) | 15.7 | 9 | 501ASN(D21) - 75 GLU(OE2) | 36 |
| 10 | 501ASN(D21) - 75 GLU(OE2) | 11.9 | 10 | 494GLN(E21) - 38 GLU(OE2) | 44.2 | 10 | 501ASN(D21) - 72 PHE( O ) | 18.1 |
| 11 | 501ASN(D21) - 75 GLU(OE1) | 17.7 | 11 | 494GLN(E21) - 38 GLU(OE1) | 49 | 11 | 498CYS( H ) - 35 GLU(OE2) | 57.1 |
| 12 | 500THR(HG1) - 75 GLU(OE2) | 32.2 | 12 | 494GLN(E21) - 35 HIS(ND1) | 78.4 | 12 | 497PHE( H ) - 35 GLU(OE2) | 12.8 |
| 13 | 500THR(HG1) - 75 GLU(OE1) | 33.2 | 13 | 488ASN( H ) - 355GLY( O ) | 43.4 | 13 | 497PHE( H ) - 35 GLU(OE1) | 66 |
| 14 | 497PHE( H ) - 35 GLU(OE2) | 10.9 | 14 | 388ALA( H ) - 489GLN(OE1) | 25.4 | 14 | 496GLY( H ) - 35 GLU(OE2) | 16 |
| 15 | 497PHE( H ) - 35 GLU(OE1) | 11.5 | 15 | 354LYS(HZ1) - 496TYR(OH ) | 12.2 | 15 | 496GLY( H ) - 35 GLU(OE1) | 62.7 |
| 16 | 496GLY( H ) - 35 GLU(OE2) | 44.5 | 16 | 84TYR(HH ) - 506TYR(OH ) | 10.8 | 16 | 495TYR( H ) - 38 ASP(OD2) | 32.7 |
| 17 | 496GLY( H ) - 35 GLU(OE1) | 42.7 | 17 | 84TYR(HH ) - 503GLY( N ) | 19.1 | 17 | 495TYR( H ) - 38 ASP(OD1) | 27.8 |
| 18 | 494SER(HG ) - 30 ASP(OD2) | 32.6 | 18 | 42TYR(HH ) - 490TYR(OH ) | 10.2 | 18 | 494SER(HG ) - 38 ASP(OD2) | 15.1 |
| 19 | 494SER(HG ) - 30 ASP(OD1) | 30.1 | 19 | 32LYS(HZ1) - 497GLY( O ) | 14.7 | 19 | 494SER(HG ) - 38 ASP(OD1) | 22.4 |
| 20 | 493GLN(E21) - 34 HIS(NE2) | 39.4 | 20 | 32LYS(HZ1) - 496TYR( O ) | 28.2 | 20 | 494SER(HG ) - 30 ASP(OD2) | 11.1 |
| 21 | 489TYR(HH ) - 38 ASP(OD2) | 14.9 | 21 | 32LYS(HZ1) - 495SER( O ) | 13.9 | 21 | 494SER(HG ) - 30 ASP(OD1) | 10.5 |
| 22 | 489TYR(HH ) - 38 ASP(OD1) | 14 | 22 | 32LYS(HZ1) - 495SER(OG ) | 33.9 | 22 | 489TYR(HH ) - 355ASP(OD2) | 19.4 |
| 23 | 486PHE( H ) - 37 GLU(OE1) | 13.6 | 23 | 25GLN(E21) - 506TYR(O2 ) | 12.9 | 23 | 489TYR(HH ) - 355ASP(OD1) | 16.1 |
| 24 | 485GLY(H1 ) - 388GLN( O ) | 19.5 |  |  |  | 24 | 487ASN(D21) - 355ASP(OD2) | 19.8 |
| 25 | 485GLY(H1 ) - 386ALA( O ) | 37.5 |  |  |  | 25 | 487ASN(D21) - 355ASP(OD1) | 21.8 |
| 26 | 485GLY(H1 ) - 352GLY( O ) | 31.7 |  |  |  | 26 | 487ASN( H ) - 354GLY( O ) | 42.6 |
| 27 | 354GLY( H ) - 485GLY( O ) | 23.1 |  |  |  | 27 | 486PHE( H ) - 385TYR( O ) | 16.5 |
| 28 | 353LYS(HZ1) - 495TYR(OH ) | 11.5 |  |  |  | 28 | 485GLY(H1 ) - 383MET( O ) | 14.8 |
| 29 | 353LYS(HZ1) - 488ASN( O ) | 16 |  |  |  | 29 | 76GLN(E21) - 501ASN(OD1) | 30 |
| 30 | 353LYS(HZ1) - 486PHE( O ) | 17.2 |  |  |  | 30 | 76GLN(E21) - 500THR(OG1) | 18.5 |
| 31 | 76GLN(E21) - 501ASN(OD1) | 57.4 |  |  |  | 31 | 42GLN(E21) - 495TYR(OH ) | 40.9 |
| 32 | 68LYS(HZ1) - 504GLY( O ) | 10.5 |  |  |  | 32 | 31LYS(HZ1) - 497PHE( O ) | 12.9 |
| 33 | 34HIS(HD1) - 494SER(OG ) | 10.9 |  |  |  | 33 | 31LYS(HZ1) - 496GLY( O ) | 22.6 |

Figure legend:

Figure S1- The overall (A) and detailed (B) illustrations of full-length ACE2 enzyme in complex with SARS-CoV-2 RBD. ACE2, B0AT1 transporter, and RBD are depicted as dark blue, green cyan, and pink, respectively. the overall structure was obtained by superimposing 6m0j and

Figure S2- (A) the superimposition of the designed peptides and positions 4 and 14 on PD. (B)The alignment of peptides’ sequences.

Figure S3- the Rg (A), SASA (B), and RMSF (C) plots of P23 (green), P28 (red), and P31(orange).

Figure S4- The validation of the inhibitory potential of designed peptides by molecular docking simulation of P23 (green), P28 (red), and P31 (orange)-bound PD (dark blue) and Wuhan RBD.

Figure S5- The validation of the inhibitory potential of designed peptides by molecular docking simulation of P23 (A), P28 (B), and P31 (C) against RBDs of beta, gamma, delta, and omicron variants. The RBM motif of RBD (light pink) is indicated in hot pink.

Figure S6- The changing number of hydrogen bonds in P23(A), P28(B), and P31(C)-PDB interfaces during the simulation.

Figure S7- The MD trajectory analyses of P4 (blue) and P68(violet) in terms of RMSD (A), Rg (B), SASA (C), number of hydrogen bonds (D), and RMSF (F).

Figure S7- the change in mobility of PD residues upon binding of the least (green) and the most (red) potent inhibitors. The residues that both groups influenced are indicated as blue.
