# Supplementary figures and images for: Design of novel disturbing peptides against ACE2 SARS-CoV-2 spike-binding region by computational approaches
Source: Front Pharmacol. 2022 Nov 11;13:996005. doi: 10.3389/fphar.2022.996005 (PMC9692113; doi:10.3389/fphar.2022.996005)

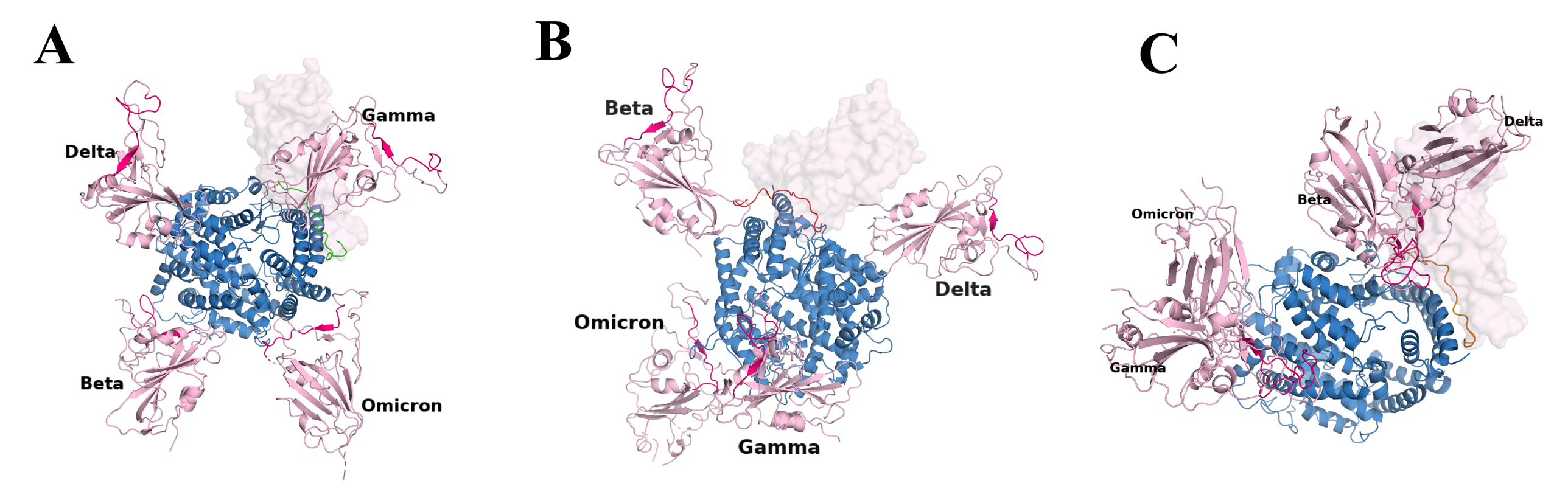

Supplement: Supplementary file 2 [file Image5.PNG]

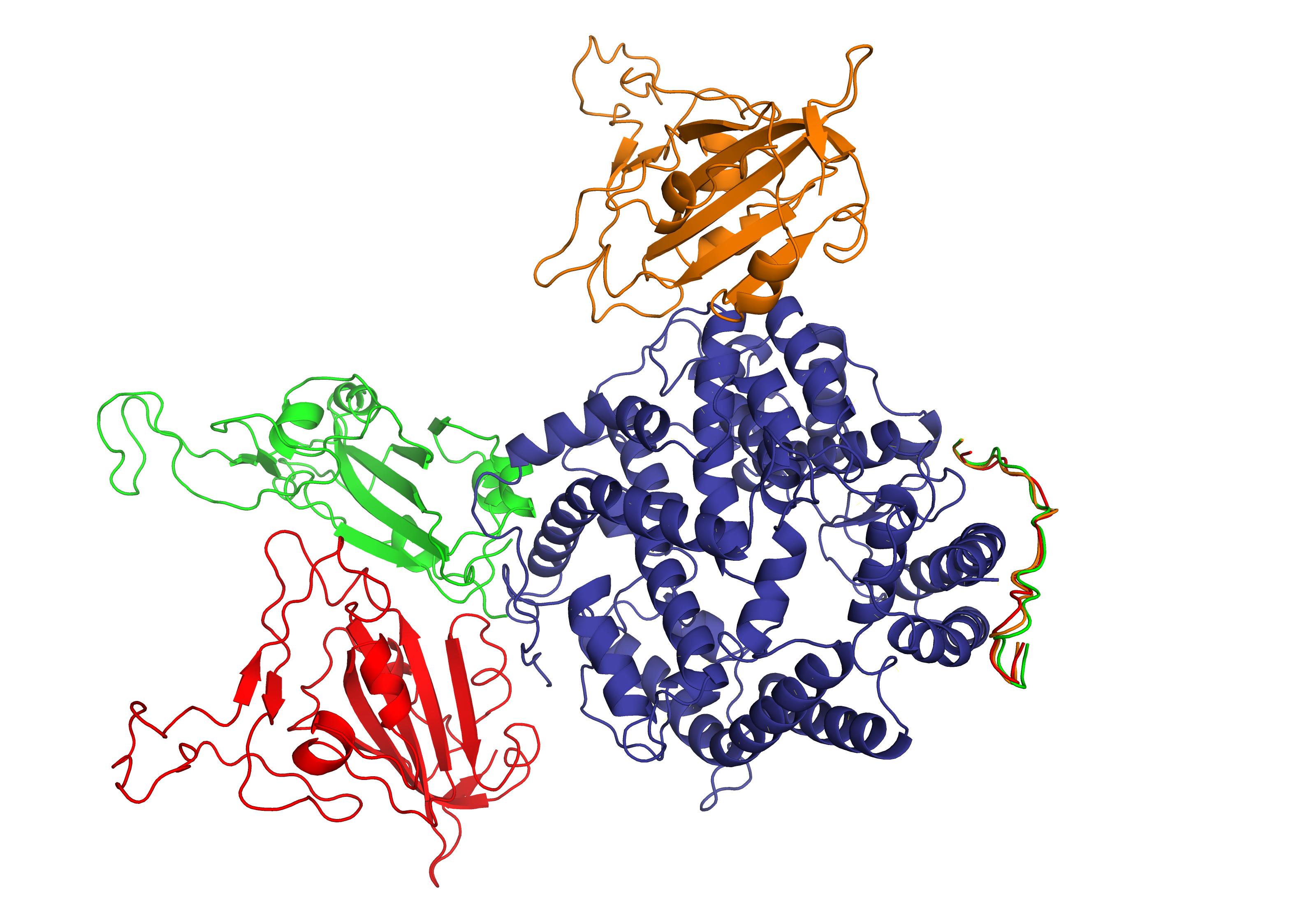

Supplement: Supplementary file 3 [file Image4.PNG]

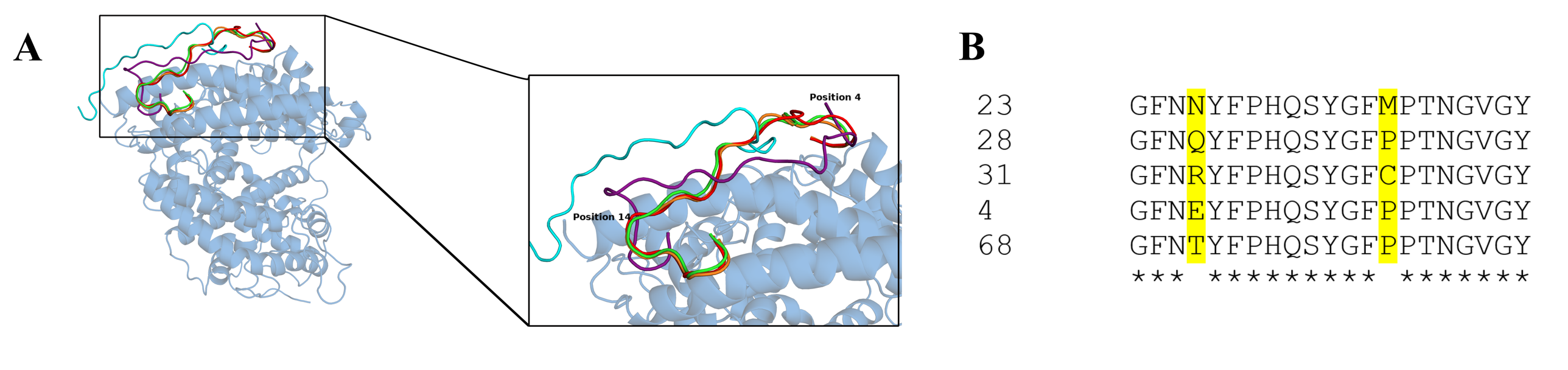

Supplement: Supplementary file 5 [file Image2.PNG]

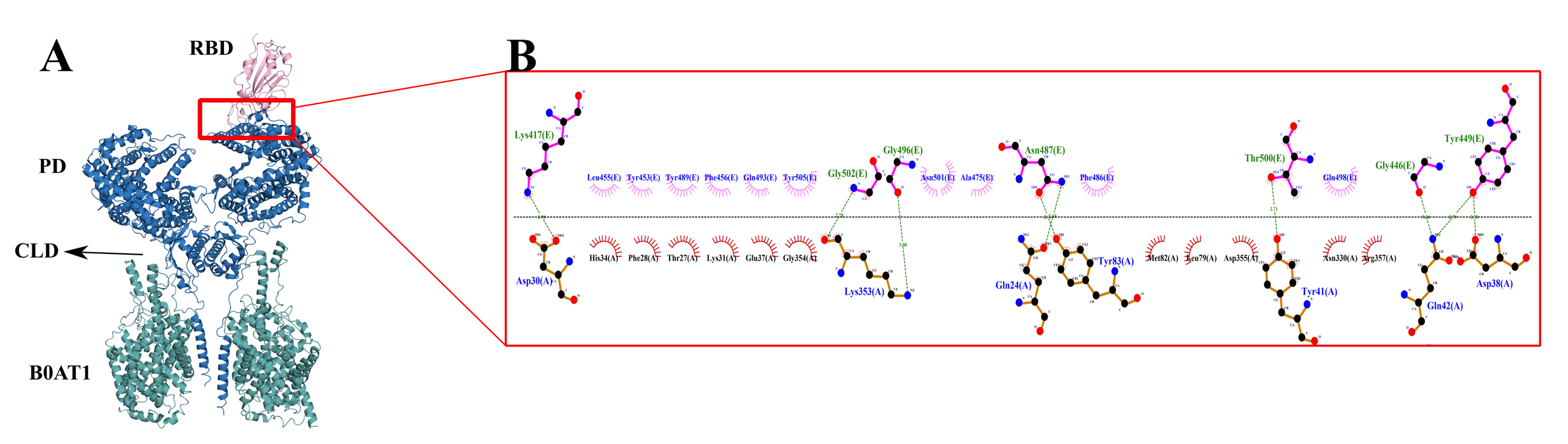

Supplement: Supplementary file 6 [file Image1.PNG]

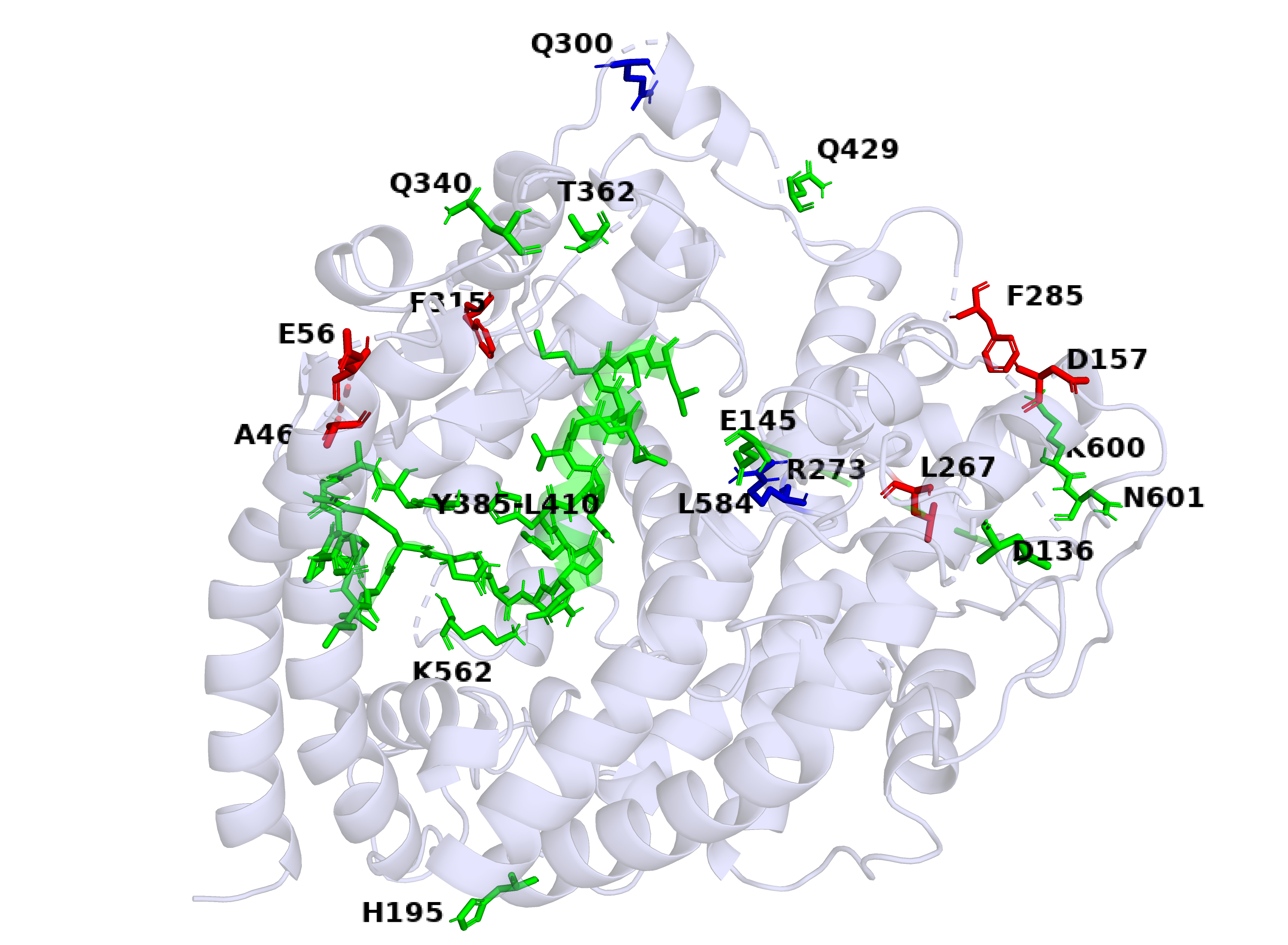

Supplement: Supplementary file 7 [file Image8.PNG]

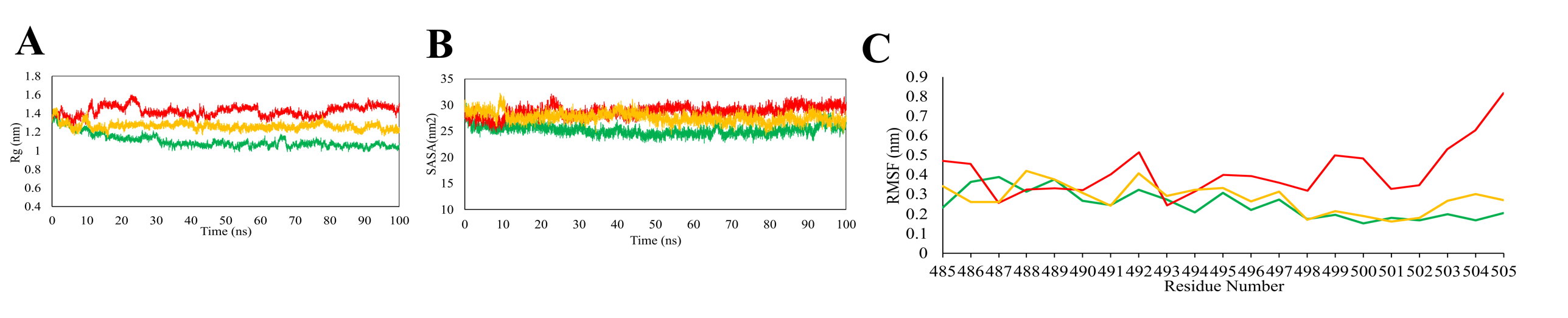

Supplement: Supplementary file 8 [file Image3.PNG]

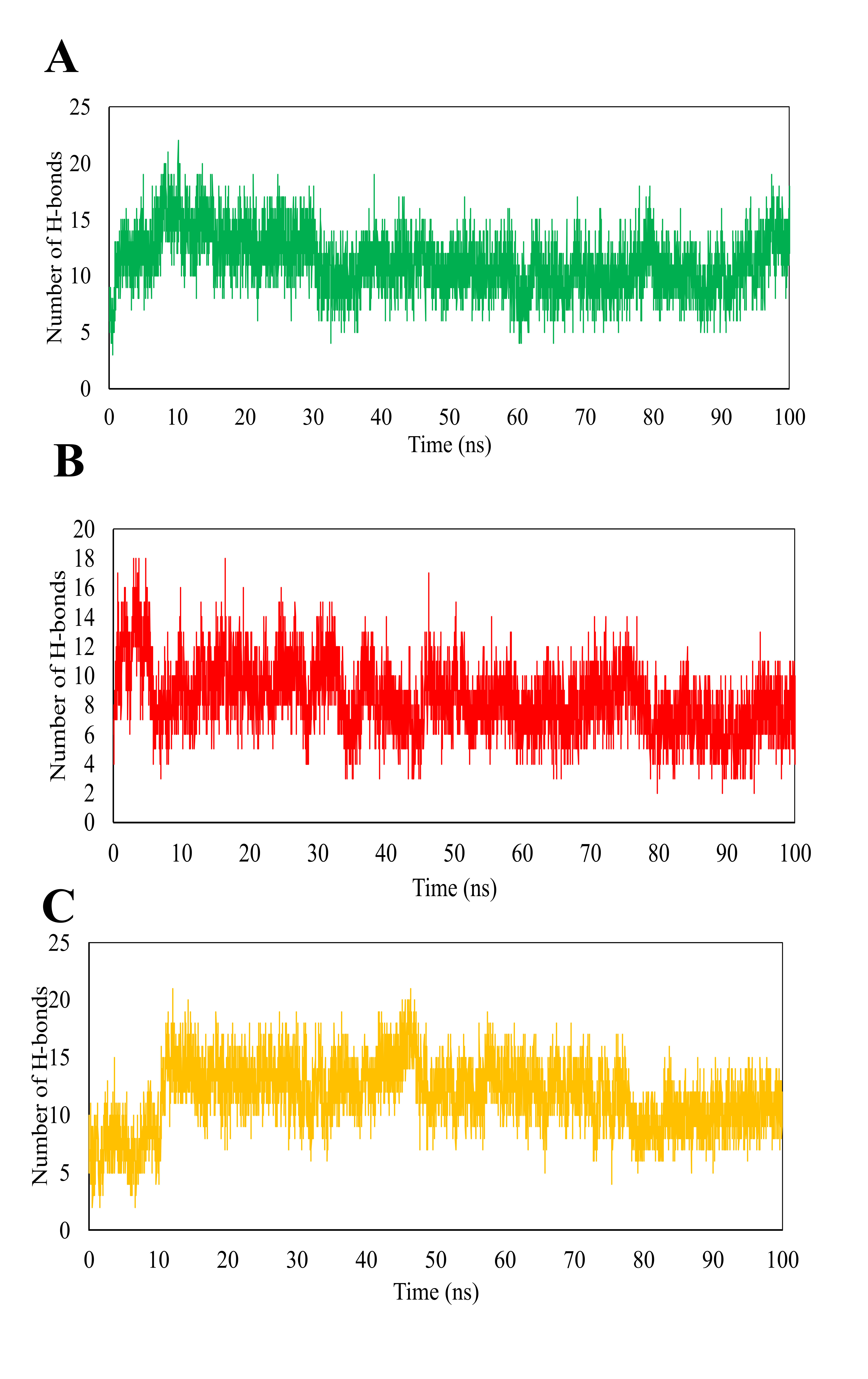

Supplement: Supplementary file 9 [file Image6.JPEG]
